# The Hypertensive Disorders of Pregnancy: A Focus on Definitions for Clinical Nephrologists

**DOI:** 10.3390/jcm11123420

**Published:** 2022-06-14

**Authors:** Elisa Longhitano, Rossella Siligato, Massimo Torreggiani, Rossella Attini, Bianca Masturzo, Viola Casula, Ida Matarazzo, Gianfranca Cabiddu, Domenico Santoro, Elisabetta Versino, Giorgina Barbara Piccoli

**Affiliations:** 1 Néphrologie et Dialyse, Centre Hospitalier Le Mans, 194 Avenue Rubillard, 72037 Le Mans, France; elisa.longhitano@libero.it (E.L.); rossellasiligato@gmail.com (R.S.); maxtorreggiani@hotmail.com (M.T.); ida.matarazzo@gmail.com (I.M.); 2 Unit of Nephrology and Dialysis, Department of Clinical and Experimental Medicine, A.O.U. “G. Martino”, University of Messina, 98125 Messina, Italy; domenico.santoro@unime.it; 3 Unit of Nephrology, Azienda Ospedaliera Universitaria Sant’Anna, 44124 Ferrara, Italy; 4 Department of Obstetrics and Gynecology, Città della Salute e della Scienza, Ospedale Sant’Anna, University of Torino, 10126 Torino, Italy; rossella.attini@gmail.com (R.A.); viola.casula@tiscali.it (V.C.); 5 Department of Obstetrics and Gynaecology, Ospedale Degli Infermi, 13875 Biella, Italy; bmast36367@yahoo.it; 6 Unit of Nephrology, Department of Advanced Medical and Surgical Sciences, University of Campania “Luigi Vanvitelli”, 80138 Naples, Italy; 7 Nephrology, Azienda Ospedaliera Brotzu, 09047 Cagliari, Italy; cabiddugianfranca@gmail.com; 8 Department of Clinical and Biological Sciences, University of Torino, 10064 Torino, Italy; elisabetta.versino@unito.it; 9 University Centre of Biostatistics, Epidemiology and Public Health, University of Torino, 10064 Torino, Italy

**Keywords:** pregnancy, chronic kidney disease, preeclampsia, small for gestational age, hypertension

## Abstract

About 5–10% of pregnancies are complicated by one of the hypertensive disorders of pregnancy. The women who experience these disorders have a greater risk of having or developing kidney diseases than women with normotensive pregnancies. While international guidelines do not provide clear indications for a nephrology work-up after pregnancy, this is increasingly being advised by nephrology societies. The definitions of the hypertensive disorders of pregnancy have changed greatly in recent years. The objective of this short review is to gather and comment upon the main definitions of the hypertensive disorders of pregnancy as a support for nephrologists, who are increasingly involved in the short- and long-term management of women with these disorders.

## 1. Introduction

The hypertensive disorders of pregnancy (HDP) are no longer seen as “transitory diseases cured by delivery”, but as windows into the future that allow us to predict a woman’s future cardiovascular and kidney health.

This paradigm shift has led to an increased need for integrated care for women who experience a hypertensive disorder during pregnancy, involving specialists that can oversee and coordinate treatment, and, whenever possible, work to identify risk factors and correct them [1,2].

Definitions of the hypertensive disorders of pregnancy have changed greatly in recent years. The objective of this article is to briefly review their main definitions and epidemiology as a support for nephrologists, who are increasingly involved in the management of HDP in the short and long term. In order to accomplish our task, we reviewed the available guidelines and the relevant literature.

## 2. The Hypertensive Disorders of Pregnancy

The definition of HDP encompasses a spectrum of conditions that extends from gestational hypertension to preeclampsia (PE), eclampsia, and to hemolysis, elevated liver enzymes, and low platelet-count syndrome (HELLP).

Other anomalies are non-uniformly aggregated in this family of diseases; this is the case for isolated or pregnancy-induced proteinuria (in normotensive pregnancies) and fetal growth restriction (FGR), which have, however, been demonstrated to predict the risk of developing PE during gestation as well as the risk of adverse short- and long-term maternal-fetal outcomes, including the development of chronic kidney disease (CKD).

The incidence of hypertensive disorders during pregnancy has increased in the last three decades, reaching 18,080,000 cases/year (a 10.92% increase), with a higher rate in South Asia and sub-Saharan Africa, in contrast to Australasia, Oceania, and Central Europe, where the lowest incidence is found, according to Wang and his colleagues in their population-based study [3]. Despite the growing number of cases, the number of deaths, estimated at 27,830 per year, has decreased significantly (a 30.05% reduction over 30 years, from 1990 to 2019); however, HDP-related mortality and morbidity remain unacceptably high [3].

There are two peaks in the age distribution of HDP, corresponding to the extremes of reproductive age [3]. The highest risk of all adverse pregnancy outcomes (including low birth weight, preterm delivery, and PE) recorded in younger age groups is influenced by several factors associated with teenage pregnancy, including low educational level, low income, malnutrition, precarious health before gestation, and marital status, factors that also reflect a continued lack of concern for young women’s health [4,5].

The other extreme of the spectrum is represented by advanced maternal age; the increase that is observed can largely be explained by hormonal changes, together with an increased prevalence of obesity, chronic diseases (mainly diabetes and hypertension), and, at least in Western countries, with recourse to medically assisted reproduction techniques [6].

## 3. Definitions

The definition of HDP has changed considerably over time; it is only recently that the principal obstetrical and gynecological international boards have agreed on diagnostic criteria, the main features of which are summarized in Table 1 [7,8,9,10,11,12].

The different conditions will be discussed in detail in the pages that follow.

### 3.1. Hypertension in Pregnancy

Gestational or pregnancy-induced hypertension, which affects up to 10% of pregnant women, is recognized by all societies as new-onset blood pressure (BP) ≥ 140/90 mmHg at or after 20 gestational weeks. The risk of progression to PE is estimated as 17.1–25% [7,8,9,10,11,12,13]. About 50% of patients with gestational hypertension continue to have high BP levels after delivery. Conversely, about 10% of the women who are normotensive during pregnancy may develop hypertension up to 6 weeks postpartum [14]. In the latter case, the patients have the same risk of short- and long-term cardiovascular complications as women affected by hypertension during gestation [15].

While the prevalence of chronic hypertension increases with age, it can affect all patients of childbearing age, both in its idiopathic and secondary forms, and is estimated to be present in 1–2% of pregnancies [16]. Apart from the specific susceptibility of women of Afro-American ethnicity, the prevalence of chronic hypertension in pregnancy is rising, as shown by a population-based cross-sectional study of more than 150 million hospital deliveries in the United States, where the increase in chronic hypertension was closely related to increased maternal age and body mass index (BMI) [17]. The clinical relevance of chronic hypertension in pregnancy is high, as it is associated with a higher rate of maternal and neonatal complications, including the need for cesarean section (41%), small-for-gestational-age (SGA) newborns (17%), preterm delivery (28%), the need for a neonatal intensive care unit (NICU) (21%), and perinatal death (4%) [18]. In addition, hypertension is an independent risk factor for “superimposed” PE (relative risk (RR), 5.1; 95% CI, 4.0–6.5) [16,19,20,21], defined by the development of proteinuria or end-organ damage in hypertensive patients after 20 gestational weeks.

Several issues are not yet clear: the blood pressure target is still a matter of discussion and, while reaching lower targets was not associated with an improvement in maternal and fetal health, the presence of known chronic kidney disease (CKD) is acknowledged as a reason for trying to reach lower BP targets (Table 2). Things are not as simple as they may seem, considering that CKD is probably the most common cause of secondary hypertension in a non-obese, non-diabetic young woman, and that many forms of CKD are not detected unless specifically searched for using both biochemical assessment and renal imaging [22,23].

Overall, the current guidelines on hypertension in pregnancy recommend treating all cases of severe hypertension (>160/110 mmHg) and suggest starting antihypertensive treatment in milder cases (BP between 140–159/90–109 mmHg) only in the presence of cardiovascular diseases, diabetes, CKD or acknowledged risk factors for cardiovascular diseases (Table 2). Once more, nephrologists often have a different view of the situation, as is suggested by the best-practice guidelines of the Italian Society of Nephrology, which, while acknowledging the risks of hypotension in pregnancy, recommend the correction of hypertension, as in the case of young patients outside the context of pregnancy (target BP ≤ 130/80 mmHg), under strict clinical surveillance [24]. The main risks to be avoided are seen as those related to hypercorrection, since hypotension can cause placental hypoperfusion, with detrimental effects on the fetus. Careful follow-up is needed to allow optimal blood pressure to be reached [24]. The issue is similar to those problems that may arise in attempting to control diabetes, where hypercorrection and frequent hypoglycemia can have negative effects on pregnancy outcomes [25]. When setting BP goals in pregnancy, we should keep in mind that outside the context of pregnancy, the results for CKD patients under tight hypertension control in the recent ACCORD BP trial demonstrated that the benefits obtained in reducing cardiovascular events and mortality were offset by a higher risk of adverse kidney outcomes, which are mainly represented by AKI [26]. No such data are available for pregnancy.

**Table 2:** Indications for starting antihypertensive medication in pregnancy, and blood pressure goals in the hypertensive disorders of pregnancy in national and international guidelines.

Guideline	Recommended Medication Initiation (mmHg)	Target BP (mmHg)
**SOMANZ, 2014 [9]**	SBP ≥ 160	<160
	DBP ≥ 110	<110
**DGGG, 2015 [27] Royal College of Physicians of Ireland, 2019 [28]**	SBP ≥ 150	<150
	DBP ≥ 100	80–100
**Brazilian Guideline of Arterial Hypertension, 2016 [29]**	SBP > 150	130–150
	DBP > 100	80–100
**Queensland, 2016 [30]**	SBP ≥ 140	<140
	DBP ≥ 90	<90
**ISSHP, 2018 [7]**	SBP ≥ 140	110–140
	DBP ≥ 90	85
**Hypertension Canada, 2018 [31]**	SBP ≥ 140	DBP < 85
	DBP ≥ 90	
**ESC/ESH, 2018 [32]**	SBP ≥ 150	<140
	DBP ≥ 95	<90
**ACOG, 2020 [11]**	SBP ≥ 160	<160
	DBP ≥ 110	<110
**NICE, 2019 [10]**	SBP ≥ 140	135
	DBP ≥ 90	85

Legend: ACOG, American College of Obstetricians and Gynecologists; ACC/AHA, American College of Cardiology/American Heart Association; DGGG, Deutsche Gesellschaft für Gynäkologie und Geburtshilfe; ESC/ESH, European Society of Cardiology/European Society of Hypertension; FIGO, The International Federation of Gynecology and Obstetrics; ISSHP, International Society for the Study of Hypertension in Pregnancy; NICE, National Institute for Health and Care Excellence; Ireland, SOMANZ, Society of Obstetric Medicine of Australia and New Zealand; Queensland, Queensland Clinical Guideline. SBP, systolic blood pressure; DBP, diastolic blood pressure.

### 3.2. Preeclampsia

**Figure 1:**
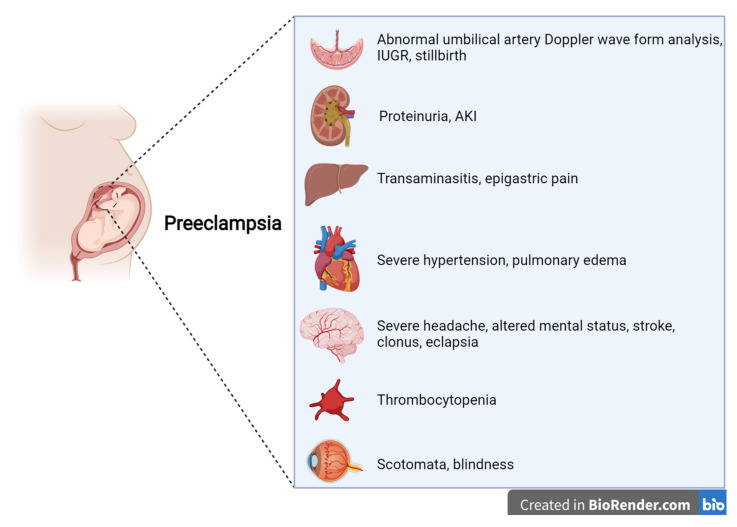
Features of preeclampsia, according to international guidelines.

PE is a multisystem disease, generally defined as a condition in which the onset of arterial hypertension after 20 weeks of gestation is accompanied by at least one sign of renal, hepatic, central nervous system, or hematologic impairment [7,8,9,10,11,12] (Figure 1). Proteinuria levels ≥0.3 g/24 h or a protein-to-creatinine ratio (PCR) ≥ 0.3 g/g is present in about 75% of the cases defined as PE. While, in the past, proteinuria was required for a PE diagnosis, international boards now consider it to be of the same importance as other symptoms and signs [7,8,9,10,11,12]. In this regard, the new guidelines underline the central role of kidney involvement, basing a diagnosis not only on proteinuria but also on a reduction in kidney function from the baseline pre-gestational level [7,8,9,10,11,12].

Other “new” features of the definition of PE are laboratory findings regarding liver involvement, with an increase in aminotransferases (alanine aminotransferase and/or aspartate aminotransferase at >40 IU/L, whether or not this is accompanied by right-upper quadrant or epigastric abdominal pain), thrombocytopenia (platelets at <10^9^/L), disseminated intravascular coagulation (DIC), and hemolysis. In this regard, the more extensive PE definition merges with the definition of HELLP syndrome, indirectly alluding to a univocal definition of HDPs.

**Figure 2:**
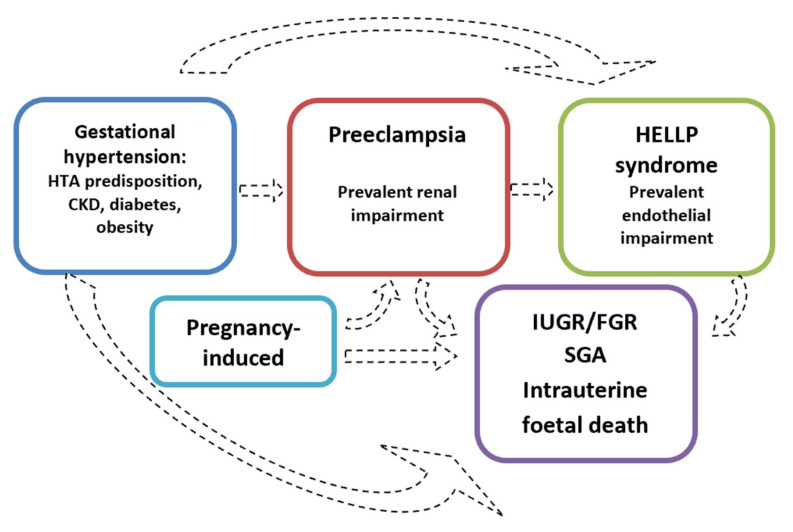
The hypothesis of distinct disorders that may merge into one another.

Symptoms reported by patients range from severe headaches, persistent visual scotomata, or blindness to alterations in mental state and complications such as clonus, eclampsia, and stroke. The Society of Obstetricians and Gynaecologists of Canada (SOGC) and the Society of Obstetric Medicine of Australia and New Zealand (SOMANZ) also include cardiac and/or pulmonary involvement in the diagnostic criteria for PE [7,8,9,10,11,12]. Moreover, except for the American College of Obstetrics and Gynecologists (ACOG), all other societies include signs of uteroplacental dysfunction, such as fetal growth restriction, an abnormal umbilical artery Doppler wave, and stillbirth as being diagnostic criteria of PE [7,8,9,10,12]. Once more, this choice reflects the view that all HDPs are interlinked, even though they do not necessarily occur with the same severity (Figure 2).

A recent retrospective cohort study of 22,094 pregnancies, followed up at Monash Health in Australia, showed that the wider criteria for defining PE, such as those adopted by the International Society for the Study of Hypertension in Pregnancy (ISSHP) in 2018, made it possible to classify a further 14.8% of women, who in the past would have been excluded from consideration by the ISSHP’s 2018 or ACOG’s 2018 guidelines, as having experienced PE [7,11,33]. The ISSPH’s 2018 guidelines highlighted that among the signs included for the diagnosis of PE, FGR, thrombocytopenia, proteinuria, AKI, or liver impairment had the strongest association with the risks of severe adverse maternal-fetal outcomes [33].

This acknowledgment of the high heterogeneity of PE, in parallel with the proposal to unify the pathogenesis of PE and other hypertensive disorders of pregnancy, has led to an attempt to stratify PE, based on a variety of elements from severity to the period of onset and pathogenic mechanisms (Table 3) [34]. While none of these has proved to be sufficient to clearly distinguish between phenotypes and, above all, to be invariably correlated with maternal-fetal short- and long-term outcomes, these approaches are the basis of a better description of the phenotype of PE, which, albeit with wide overlaps, can enable clinicians to perform a prognostic evaluation of individual cases. In fact, even though late-onset PE may be life-threatening for both mother and fetus, early-onset PE is more often “placental”, severe, and associated with profound angiogenic-antiangiogenic imbalance, and, at least according to some studies, has a higher risk of recurrence in subsequent pregnancies [35,36,37].

### 3.3. Small for Gestational Age, Intrauterine Growth Restriction, Fetal Growth Restriction (FGR), and Intrauterine Death

The hypertensive disorders of pregnancy have been related to defective placentation, which may be severe enough to impair the physiologic development of the fetus during pregnancy. These include babies that are small for gestational age (SGA), intrauterine growth restriction (IUGR) or its synonymous fetal growth restriction (FGR), and an increased risk of pre-term delivery, which is usually classified as very early pre-term (<28 gestational weeks), early pre-term (28–34 weeks or, presently, more often, 28–32 gestational weeks) and late pre-term (according to the previous definitions: 32–37 or 34–37 gestational weeks), or intrauterine death [7,8,9,10,11,12]. In this scenario, FGR, newborns that are small for gestational age, and intrauterine death, are part of the spectrum of the hypertensive disorders of pregnancy; while they can occur in association with hypertension or full-blown PE, they may also be isolated and without other PE features [7,8,9,10,11,12].

FGR is defined by the fetal weight, or an abdominal circumference estimated using ultrasounds, showing growth between the 3rd and 10th percentile for gestational age (moderate fetal growth restriction) or less than the 3rd percentile (the severe form) [38]. However, other studies set the cut-off points at the 10th and 5th percentile. The definition of FGR is also dynamic and may need to be raised when there is a flattening of the growth curve [38], to distinguish between low-birth-weight babies with harmonic growth, for genetic (or pathological) reasons, and babies whose normal growth was impaired at some point in their intrauterine life. Considering that FGR can also be the result of fetal diseases, a genetic evaluation should be proposed by the obstetric team, in cases of early recognition (<32 gestational weeks) or when in association with polyhydramnios or, more obviously, of fetal malformations [38].

Conversely, the definition of small for gestational age (SGA) usually applies if the effective birth weight is less than the 10th percentile of the expected adjusted figure for gestational age, according to local growth curves; once more, the definition is not homogeneous, and the cut-off point of less than the 5th percentile is also used [38].

The International Society of Ultrasound in Obstetrics and Gynecology (ISUOG) guidelines distinguish between FGR and SGA, as follows: FGR is defined as the fetus failing to reach its genetically predetermined growth potential, while SGA is diagnosed when the fetus size falls below a predefined threshold for its gestational age (usually the 10th percentile) [39]. In addition, these guidelines specify that an SGA fetus may be small but not at increased risk of adverse perinatal outcomes, while a fetus with a size above the 10th percentile may still be considered FGR and at increased risk of adverse perinatal and long-term outcomes [39].

Intrauterine death is distinguished from miscarriage, which is defined as fetal loss before 20 gestational weeks in the United States, while the British guidelines still set the cut-off point at 24 weeks, which was previously the agreed point. The anticipation of the definition is due to current improvements in allowing viability in very preterm babies, as the definition of intrauterine death usually applies to gestational ages compatible with survival [40,41,42].

According to the US fetal mortality rate, in 2013, “early” intrauterine death was estimated as occurring in 3.01 per 1000 births before 28 gestational weeks, while “late” cases (after 28 weeks) occurred in 2.97 per 1000 births [43]. Over 30% of these fetuses were SGA, often without a causal diagnosis [44].

The relevance for nephrologists of the inclusion of FGR in the context of the hypertensive disorders of pregnancy is high. Including these conditions increases the number of cases that, at least according to the Italian Society of Nephrology (to date, the only group to have published a specific statement on follow-up after preeclampsia), should undergo a basic nephrology evaluation to exclude the presence of CKD [45].

The probability that PE is associated with pre-existing CKD is high, as has also been reported by the Mayo clinic, and it is probably present in at least 20% of the women who experience a PE episode [22,45,46].

The second element of interest is that being born small for gestational age and/or with a low birth weight is, in turn, associated with the risk of developing CKD, hypertension, diabetes, and metabolic syndrome in adulthood and, for females, with developing preeclampsia once pregnant, thus generating a vicious circle of diseases reappearing over generations, not necessarily with a genetic basis [47,48,49,50]. Despite the fact that the relationship between low birth weight and prematurity and subsequent disorders during pregnancy has been described for decades, the message that the risk of preeclampsia in the mother decreases with increased gestational age has not been fully integrated into clinical practice, and no specific surveillance programs have been set up for such high-risk pregnancies (the odds ratio (OR) of developing PE was about 4 for mothers born earlier than 34 gestational weeks, and is even higher for those born under 4.5 lb, about 2 kg) [47].

Among the numerous subsequent studies on this important issue, the recent large population study by Gjerde and co-workers deserves mention: considering over two million individuals, after a mean follow-up period of 26 years, low birth weight was associated with an odds ratio of 1.72 for CKD, 1.79 for SGA, and 1.48 for preterm birth (95% CI, 1.33 to 1.66) [51].

### 3.4. HELLP Syndrome

**Figure 3:**
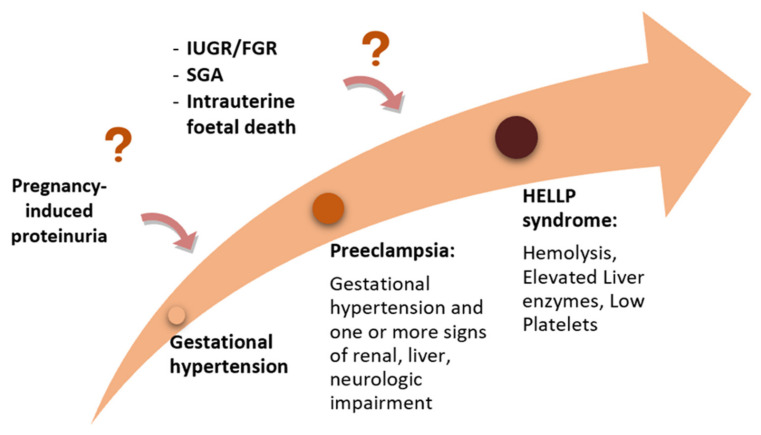
The “sequential hypothesis” of a continuum of severity of the hypertensive disorders of pregnancy.

The position of the HELLP syndrome in the context of the hypertensive disorders of pregnancy is still debated, although this severe condition is currently included in most guidelines regarding HDP [7,8,9,10,11,12]. Currently, there are two different approaches: one that considers HELLP syndrome a severe variant of PE (Figure 3), and one that sees it as a distinct disease, while acknowledging that there is a shared pathogenesis and risk factors as well as an interplay between all these disorders [34].

Along with hemolysis, thrombocytopenia, and liver impairment, other typical features are hypertension, present in 82–88% of pregnant women, and proteinuria, which is found in 86–100% of pregnant women, according to the cohorts evaluated [52].

Conversely, an interesting “unifying” approach includes HELLP syndrome, together with pregnancy-induced thrombotic microangiopathies (TMAs), mainly because they share patterns of endothelial cell injury (primarily affecting the kidneys and the cardiovascular and central nervous systems, with a hemolytic uremic syndrome, thrombotic thrombocytopenic purpura, and catastrophic antiphospholipid syndrome) [53]. Laboratory findings, including peripheral thrombocytopenia, mechanical hemolytic anemia (defined by hemoglobin levels of ≤10 g/dL, lactate dehydrogenase (LDH) at the upper limit of normal, undetectable haptoglobin, and the presence of schistocytes on the blood smear), as well as signs of impaired organ function, such as a rise in serum creatinine or transaminases, can be present in PE and HELLP as well as in TMAs [53]. Although this interpretation is not universally accepted, it offers a change in perspective, making it possible for us to see the hypertensive disorders of pregnancy not only as “transient” alterations due to gestation but also as endothelial disorders that may recur in subsequent pregnancies or evolve into long term endothelial dysfunction and, in turn, CKD.

### 3.5. Acute Kidney Injury in Pregnancy

The definition of acute kidney injury (AKI) in pregnancy, in the absence of agreed formulae for calculating the kidney function, is usually based on serum creatinine (the definition of AKI usually corresponds to an increase in serum creatinine of at least 0.3 mg/dL over baseline) [54]. However, the physiological reduction in serum creatinine during physiological pregnancies should be considered. In this context, two recent systematic reviews suggest that a normal serum creatinine value in pregnancy should correspond to less than 80% of the pre-pregnancy normal one [55,56]. However, since serum creatinine assessment is not part of the work-up of physiological pregnancies, the interpretation of a serum creatinine level that is still in the normal range in pregnancy may be difficult, if not impossible. This limitation, and the importance accorded to kidney function in pregnancy, are a further indication of the need for including at least serum creatinine in the routine control tests in pregnancy [57,58].

Moreover, we should consider that renal damage may be induced by causes other than the hypertensive disorders of pregnancy, such as hypovolemia, ischemia (e.g., severe postpartum hemorrhage), or sepsis, which can cause acute tubular necrosis or cortical necrosis with adverse outcomes for maternal renal function, both immediately and in the long term [34,59].

## 4. The Hypertensive Disorders of Pregnancy and the Future Risk of CKD and End-Stage Renal Disease (ESRD)

If, on the one hand, HDPs may reveal the presence of an underlying disease or predisposition, on the other hand, PE may act as a hit, often in the context of the multiple-hit pathogenesis of cardiovascular, renal, and metabolic disorders [60,61,62,63,64,65]. While a discussion of the cardiovascular effects of PE is beyond the scope of this review, its effects on kidney function deserve mention. After Viske’s pivotal study on the Norway registry, highlighting the risk of end-stage kidney disease after PE (an OR of about 4.7), attention has increasingly been focused on these issues: subsequent studies showed a close association between an episode of PE and the need for a kidney biopsy later in life, suggesting that hypertensive disorder may be either a trigger for or an early marker of renal dysfunction [60,66,67].

A large meta-analysis evaluating trials with at least 20 preeclampsia patients, with a follow-up of ≥4 years, involving 110,803 PE cases and 2,680,929 controls, quantified the OR of developing ESRD after preeclampsia at 6.35; however, it also suggested a knowledge gap regarding the “intermediate” phases of CKD, thus indicating the need for prospective follow-up after PE, to better clarify the natural history of CKD after PE [68]. Of note, the baseline CKD risk is probably significant, as shown by the different risk curves observed in Norway (low incidence of CKD) and Taiwan (high incidence of CKD) [69].

Moreover, the best way to manage patients after an episode of pregnancy-related AKI remains undefined [1,34,70]. The hypertensive disorders of pregnancy, in particular PE and HELLP, can further exert a negative effect on kidney health via the development of AKI; once more, the baseline renal conditions matter, and especially in developing countries, the difference between AKI and AKI associated with CKD may be difficult to distinguish, but it is relevant to the future of renal function, i.e., 7–29% of the women needing dialysis in pregnancy fail to fully recover [71,72,73,74].

## 5. Risk Factors for PE and HDP

A long-standing misconception is that PE more frequently, or even almost exclusively, affects first pregnancies. Things are not so simple; the incidence of PE in subsequent pregnancies is an example of conditioned probability. In fact, as reported in a classic study on a large cohort, conducted by Hernandez Diaz and co-workers in Sweden in 2009, the risk of PE was found to be 4.1% in first pregnancies, while the incidence of PE was 1.7% in the subsequent gestations of patients without a history of PE, and PE in the first pregnancy was associated with a 14.7% risk of its recurrence in the second gestation and a risk of up to 31.9% after two PE episodes [75]. While these figures hold true for singleton pregnancies, the risk of a first PE episode is higher in multiple pregnancies, where the probability of developing PE was estimated to be 12% in the Swedish study [74], but, interestingly, without significantly increasing the risk of recurrent PE in subsequent singleton pregnancies. The phenotype of PE also seems to modulate the risk of recurrence and, according to the same study, the risk of recurrence was much higher in early-onset PE (up to 60% in a second pregnancy and up to 90% after two early PE episodes) [75].

However, the data on the risk of recurrence varies widely: a population-based record-linkage cohort study in Australia found much lower cumulative rates of recurrence of PE in the subsequent pregnancy after a first early PE episode. Comparing early PE (<28 weeks) to PE at 28–33 weeks, the first group was found to be more likely to deliver preterm (38.8% and 28.7%, respectively) and to experience perinatal death (4.3% vs. 1.2%) during a subsequent gestation [76]. Overall, 33.8% of women who experienced an episode of early PE during the first pregnancy developed PE in a subsequent singleton pregnancy [76]. Other smaller cohort studies report a variable recurrence rate of preeclampsia, ranging from 25 to 65% [77,78,79,80].

A recent meta-analysis of 92 studies, involving 25,356,688 gestations, identified further maternal predisposing conditions for the onset of PE [19]. Interestingly, apart from having experienced a hypertensive disorder and/or placental dysfunction during pregnancy, a familial history of PE in a first-degree relative represented a significant risk factor (RR 2.90, 95% CI 1.70–4.93) [19].

According to a meta-analysis published in 2003, maternal obesity (BMI ≥ 30 kg/m^2^) confers a relative risk (RR) of 2.8 (95% CI 2.6–3.1), doubling each 5–7 kg/m^2^ of BMI and, according to the same sources, even being overweight (BMI 25–30 kg/m^2^) significantly increases the risk of PE (RR 2.1 95% CI 2.0–2.2) [81]. Conversely, advanced maternal age has a modest effect on PE (35–40 years old, RR 1.2, 95% CI 1.1–1.3; ≥40 years old, RR 1.5, 95% CI 1.2–2.0) [19]. According to the same meta-analysis, chronic hypertension confers a relative risk of PE of up to 5.1 and, as is true for obesity, this is proportional to the severity of the baseline condition, followed by pre-existent diabetes (RR 3.7), autoimmune diseases, such as antiphospholipid syndrome (RR 2.8, 95% CI 1.8–4.3) and systemic lupus erythematosus (RR 1.8, 95% CI 1.5–2.1); the latter is estimated to have the same “weight” as CKD (RR 1.8, 95% CI 1.5–2.1), the risk of which varies according to the stage [19]. Interestingly, PE predisposing factors are almost the same as those for CKD, emphasizing the link between these diseases [46].

Besides multiple pregnancies (with a relative risk estimated here of 2.9), the adoption of medically assisted reproduction should be considered the criterion for more rigorous follow-up in gestation (RR 1.8); once more, the risks of medically assisted reproduction are modulated by the type of intervention, with the greatest risk being associated with egg donation, possibly influenced by generally higher maternal age and, often, reduced baseline kidney function [82].

The elements defining high-risk and moderate-risk factors for PE in the different guidelines are summarized in Table 4.

## 6. Follow-Up Outside the Context of Pregnancy

Despite the increasing evidence of long-term risks to kidney health after PE, the only indication included in gynecological international guidelines regards the need for yearly screening of blood pressure, lipids, fasting blood sugar, and body mass index for women with prior preeclampsia that was preterm (<37 weeks) or recurrent [11,12].

The American Heart Association (AHA) has included a history of PE or gestational hypertension as a major risk factor for cardiovascular disease since 2011 [85,86] and, in a 2017 meta-analysis, Wu and colleagues confirmed that PE was independently associated with an increased risk of future heart failure (RR 4.19), coronary heart disease (RR 2.50), global cardiovascular disease-related death (RR 2.21), and stroke (RR 1.81) [87].

As for nephrology guidelines, in 2017, those of the Italian Society of Nephrology included a best-practice statement for the prevention and follow-up of PE [45]. After the first episode of a hypertensive disorder of pregnancy, a multidisciplinary basic pre-gestational screening is deemed desirable, to identify undisclosed maternal diseases and/or to make possible the timely treatment of predisposing factors, such as hormonal imbalance, obesity, and nutritional deficits [56]. It is also suggested that if recurrent or severe PE occurs in a woman with acknowledged risk factors, she should be referred to a tertiary care center in the case of subsequent gestation [45].

While this may be difficult to put into practice, given a lack of dedicated resources, the expert agreement reported in the best practice holds that a woman should be monitored by a nephrologist for at least 6 months after a PE episode (monthly, until the normalization of proteinuria and hypertension, and at least once for 2 to 3 months afterward) to identify unresolved hypertension, kidney function impairment or persistent proteinuria. In these cases, the consensus statement holds that women should be monitored according to local indications for CKD, while yearly follow-ups should be offered to other women, especially those who express the wish for a new pregnancy. Screening should include blood pressure control and the assessment of proteinuria or albuminuria, as well as kidney function. The appearance of hypertension or proteinuria should prompt additional testing to determine its cause and to guarantee an early treatment start [45].

## 7. Follow-Ups in Subsequent Pregnancies

While the correction of pre-gestational BMI and other potentially reversible predisposing factors has not been demonstrated to completely offset the increased risk of PE recurrence, it appears to mitigate the risk of adverse pregnancy-related events [56]. Dietary management before and during pregnancy should be considered in both overweight and underweight patients, as well as screening for vitamin status, in particular for the vitamin D axis, which appears to be related to PE onset [56,88,89].

Self-monitoring of blood pressure, possibly integrated as needed with 24-h blood pressure measurement, is part of the management recommended for women classified as being at high risk of PE. Similarly, starting from the 20th to the 24th week, frequent checking of the proteinuria/creatinuria ratio on spot urine (for instance, every 1–2 weeks, according to clinical condition) can allow the early identification of incipient proteinuria; monitoring the sFLT1/PIGF ratio, starting at the 20th week, can make it possible to have the elements available to anticipate placental distress [90]. Moreover, the sFlt-1 to PlGF ratio may help in differentiating between the different causes of increasing proteinuria, especially in CKD patients: a low ratio (<30), in fact, is usually associated with CKD, while a ratio of >150 is not compatible with CKD alone and is highly suggestive of preeclampsia [91]. This information may help clinicians in the management of pregnancy, prolonging gestation in the case of CKD, or intensifying care or anticipating the delivery in the presence of preeclampsia.

Low-dose acetylsalicylate (ASA) is considered to be the most promising drug for reducing the risk of PE and FGR, as well as preterm birth and perinatal mortality in women classified as being at high risk of PE [92,93,94]. According to the ASPRE trial, in fact, women taking low-dose ASA during their pregnancies had a 0.38 odds ratio of experiencing PE compared to controls [95]. There is still a lack of universal consensus on ASA dose (current recommendations range from 75 to 150 mg), and the timing of treatment start, although the idea of “the earlier the better” is widespread (Table 5) [96].

## 8. Conclusions

Research on the hypertensive disorders of pregnancy over the past 20 years has led to the recognition of these ailments as possible “signs” of an underlying disease and as “precursors” of future health problems involving the kidney.

Although pregnancy can reveal future or existing kidney disorders, which are difficult to diagnose if they are not known about and searched for, the key message of the usefulness of nephrological surveillance has not yet been standardized in common clinical practice.

Even more “neglected” than the mother is the offspring, although it is known that children with a low birth weight, which is frequently related to hypertensive disorders during the mother’s pregnancy, have a greater risk of developing chronic kidney disease and a greater risk, for girls, of future preeclampsia.

Identification and correction, where possible, of the risk factors for both these disorders and for potential future health problems, early counseling, and long-term nephrological follow-up could help improve the management of the health of women and their offspring.

Prospective longitudinal studies with large cohorts are necessary to support the key role of the kidney, and clearly define the need for clinical evaluation by a nephrologist during and after an episode of a hypertensive disorder in pregnancy.

## Figures and Tables

**Table 1:** Definitions of the hypertensive disorders of pregnancy, as found in the national and international guidelines.

Scientific Society	Chronic Hypertension	Gestational Hypertension	Preeclampsia (PE)	Super-Imposed Preeclampsia	Other Hypertensive Categories
**ACOG, (2020) [11]**	HTN diagnosed before 20 gestational weeks with 2 measurements at least 4 h apart	New-onset HTN diagnosed with 2 measurements at least 4 h apart after 20 gestational weeks	New-onset HTN between 20 gestational weeks and up to 2 weeks postpartum, with at least one of the following: proteinuria ≥ 0.3 g/24h or PCR ≥ 0.3 g/g or dipstick 2+ AKI AST or ALT doubling pulmonary edema thrombocytopenia headache with visual symptoms or unresponsive to medications	Chronic HTN and development of associated proteinuria	White-coat HTN: detection at office/clinic of BP ≥ 140/90 mmHg, but normal domiciliary blood pressure (<135/85 mmHg)
**ISSHP, (2018) [7]**	HTN diagnosed before 20 gestational weeks, with 2 measurements at least 4 h apart during the same visit or in two consecutive visits	New-onset HTN diagnosed at or after 20 gestational weeks in the absence of features of PE	Gestational hypertension with at least one of the following: proteinuria other maternal organ dysfunction, including: AKI liver involvement (ALT or AST > 40 IU/L) with or without right upper quadrant or epigastric abdominal pain neurological complications (eclampsia, altered mental status, blindness, stroke, clonus, severe headache, persistent visual scotomata) hematological complications (thrombocytopenia, DIC, hemolysis) uteroplacental dysfunction (such as fetal growth restriction, abnormal umbilical artery Doppler waveform analysis, or stillbirth)	Any of the maternal organ dysfunctions consistent with preeclampsia developing in chronic hypertensive patients or new-onset proteinuria accompanied by a rise in blood pressure	White-coat HTN: detection at office/clinic of BP ≥ 140/90 mmHg, but normal domiciliary blood pressure (<135/85 mmHg) Masked HTN: normal at a clinic or office measurement, elevated at other times, and diagnosed by 24 h ABPM or HBPM
**FIGO, (2021) [12]**	Aligned with ISSHP criteria	Aligned with ISSHP criteria	Aligned with ISSHP criteria	Aligned with ISSHP criteria	
**RCOG, (2019) [10]**	HTN diagnosed before 20 gestational weeks or in a patient already taking anti-hypertensive drugs when referred at first visit	New-onset HTN diagnosed after 20 gestational weeks, without significant proteinuria	Aligned with ISSHP criteria	Aligned with ISSHP criteria	
**SOCG, (2014) [8]**	HTN diagnosed before 20 gestational weeks associated with comorbid conditions or with superimposed PE	New-onset HTN diagnosed after 20 gestational weeks associated with comorbid conditions or with evidence of PE	Gestational HTN with at least one of the following: new-onset proteinuria, “adverse conditions” (involvement of an organ system): CNS (headache, visual symptoms, seizure, etc.) cardiorespiratory (chest pain, hypoxia, poorly controlled HTN, etc.) hematologic (thrombocytopenia, elevated INR or PTT renal (elevated uric acid, AKI) hepatic (elevated AST and/or ALT, right upper quadrant pain, hypoalbuminemia, etc.) fetoplacental system (abnormal fetal heart rate, oligohydramnios, stillbirth, etc.) “severe adverse complications”: - CNS (eclampsia, PRES, cortical blindness, Glasgow coma scale 13, stroke, TIA, or RIND) - cardiorespiratory (uncontrolled severe HTN over 12 h, oxygen saturation <90%, pulmonary edema, positive inotropic support, or myocardial ischemia or infarction) - hematologic (thrombocytopenia < 50 × 10^9^/L or transfusion of any blood product) - renal (AKI or new indication for dialysis) - hepatic (INR.2 in the absence of DIC or warfarin) - fetoplacental system (abruption with evidence of maternal or fetal compromise, reverse ductus venosus A wave, or stillbirth)	Chronic HTN with at least one of the following: - resistant HTN - new-onset or worsening of proteinuria - one or more “adverse conditions” (e.g., PE) - one or more “severe complications” (e.g., PE)	Aligned with ISSHP criteria
**SOMANZ, (2014) [9]**	HTN diagnosed before 20 gestational weeks	New-onset HTN diagnosed after 20 gestational weeks, followed by the return of pregestational levels of BP within 3 months postpartum	New-onset HTN after 20 gestational weeks and involvement of one of the following systems: renal (proteinuria, AKI) hematologic (thrombocytopenia, hemolysis, DIC) hepatic (raised ALT and AST, epigastric or right upper quadrant pain) CNS (eclampsia, hyperreflexia with sustained clonus, persistent headache, visual disturbances, PRES, stroke) pulmonary edema fetal growth restriction	Chronic HTN with at least one of the systemic signs of PE after 20 gestational weeks	

Legend: ACOG, American College of Obstetricians and Gynecologists; ACC/AHA, American College of Cardiology/American Heart Association; DGGG, Deutsche Gesellschaft für Gynäkologie und Geburtshilfe; ESC/ESH, European Society of Cardiology/European Society of Hypertension; FIGO, The International Federation of Gynecology and Obstetrics; ISSHP, International Society for the Study of Hypertension in Pregnancy; NICE, National Institute for Health and Care Excellence; RCOG, Royal College of Obstetricians and Gynecologists; SOCG, The Society of Obstetricians and Gynaecologists of Canada; SOMANZ, Society of Obstetric Medicine of Australia and New Zealand; Queensland, Queensland Clinical Guideline;. HTN, hypertension; PCR, protein/creatinine ratio; AST, aspartate aminotransferase; ALT, alanine aminotransferase; AKI, acute kidney injury; PE, preeclampsia; DIC, disseminated intravascular coagulation; ABPM, ambulatory blood pressure monitoring; HBPM, home blood pressure monitoring; INR, international normalized ratio; PTT, partial thromboplastin time; CNS, central nervous system; PRES posterior reversible encephalopathy syndrome; TIA, transient ischemic attack; RIND, reversible ischemic neurological deficit.

**Table 3:** Proposed classifications of PE according to severity, time of onset, and pathogenesis.

**Mild PE**	160/110 mmHg < BP ≤ 140/90 mmHg proteinuria < 5.0 g/24 h
**Severe PE**	Signs of impairment of: ◦ CNS ◦ liver ◦ pulmonary edema ◦ kidney, with oliguria < 500 mL/24 h ◦ uncontrolled hypertension Biochemistry: ◦ proteinuria ≥ 5 g/24 h ◦ thrombocytopenia < 10^9^/L FGR
Classification based on time of onset	
**Early PE**	Onset < 34 gestational weeks, or alternatively < 32 weeks
**Late PE**	Onset ≥ 34 gestational weeks, or alternatively ≥ 32 weeks
**Postpartum PE**	Onset after delivery
Classification based on proposed pathogenesis	
**Placental PE**	Presence of severe signs of placental malperfusion
**Maternal PE**	Onset in the presence of maternal risk factors (obesity, diabetes, CKD, hypertension)
**Angiogenic PE**	PE development with an imbalance in angiogenic factors (sFlt-1/PlGF, sEnd, etc.)
**Non-angiogenic PE**	No angiogenic imbalance
**Superimposed PE**	PE onset in women affected by HTA or CKD

Modified from [34]. Legend: PE, preeclampsia; BP, blood pressure; CNS central nervous system; FGR, fetal growth restriction; CKD, chronic kidney disease; sflt-1/PlGF, soluble fms-like tyrosine kinase 1/placental growth factor; sEnd, soluble endoglin; HTA, hypertension.

**Table 4:** PE risk factors, according to international boards.

**WHO (2011) [83]**	**High risk factors:** - previous episode of PE - multiple gestations - chronic HTA - pre-existing diabetes - renal disease - autoimmune disease
**SOMANZ (2014) [9]**	**No distinction between high or moderate risk factors:** - personal or family history of PE - nulliparity - multiple pregnancies - overweight or obesity - age ≥ 40 - SBP > 130 mmHg or DBP > 80 mmHg before 20 gestational weeks - antiphospholipid syndrome - renal disease - pre-existing diabetes - chronic autoimmune disease - inter-pregnancy interval >10 years
**Queensland risk factors (2016) [30]**	**No distinction between high or moderate risk factors:** - personal or family history of PE - multiple pregnancy - nulliparity - second-grade obesity - age > 40 - SBP > 130 mmHg or DBP > 80 mmHg at initial visit - inter-pregnancy interval >10 years - renal disease - pre-existing diabetes - chronic autoimmune disease - chronic HTA - antiphospholipid antibodies
**ACOG (2019) [84]**	**High risk factors:** - Previous episode of PE - multiple gestations - chronic HTA - pre-existing diabetes - renal disease - autoimmune disease **Moderate risk factors:** - nulliparity - obesity - family history of preeclampsia (mother or sisters) - African American ethnicity - low socioeconomic status - age > 35 - history of an SGA neonate - previous adverse pregnancy outcome - inter-pregnancy interval >10 years
**ESC/ESH (2018) [32] NICE (2019) [10] Royal College of Physicians of Ireland (2019) [28]**	**High risk factors:** - hypertensive disease during a previous pregnancy - CKD - autoimmune disease - pre-existing diabetes - chronic HTA **Moderate risk factors:** - first pregnancy - age ≥ 40 - inter-pregnancy interval >10 years - second-grade obesity at first visit - family history of preeclampsia - multiple pregnancies
**ISSHP (2018) [7]**	**High risk factors:** - history of preeclampsia - chronic HTA - pre-existing diabetes - renal disease - obesity - multiple pregnancies - antiphospholipid syndrome - assisted reproduction

Legend: ACOG, American College of Obstetricians and Gynecologists; ESC/ESH, European Society of Cardiology/European Society of Hypertension; ISSHP, International Society for the Study of Hypertension in Pregnancy; NICE, National Institute for Health and Care Excellence; SOMANZ, Society of Obstetric Medicine of Australia and New Zealand; Queensland, Queensland Clinical Guideline; WHO, World Health Organization. PE, preeclampsia; HTA, hypertension; SBP, systolic blood pressure; DBP, diastolic blood pressure; SGA, small for gestational age; CKD, chronic kidney disease.

**Table 5:** Indications for the administration of acetylsalicylic acid (ASA) in preeclampsia prevention, as found in national and international guidelines.

Guideline	ASA Daily Dose	Timing of Treatment (Weeks of Gestation)	Indication for Treatment
**WHO (2011) [83]**	75 mg	before 20	≥1 high risk factor ^a^
**SOMANZ (2014) [9]**	low dose	up to 37	moderate to high risk ^b^
**DGGG (2015) [27]**	100 mg	up to 34	
**Queensland (2016) [30]**	100 mg	before 16 to 37 or delivery	moderate to high risk ^c^
**Brazil (2016) [29]**	75–150 mg	from 12 (end not specified)	intermediate or increased risk (not specified in guidelines)
**USPSTF (2017) [97]**	81 mg	from 12 (end not specified)	≥1 high risk factor ^a^
**ACC/AHA (2017) [98]**	no specific recommendation, refer to ACOG’s previous recommendations		
**ESC/ESH (2018) [32]**	100–150 mg	from 12 to 36	high ^d^ or moderate ^e^ risk
**ISSHP (2018) [7]**	75–162 mg	from 16 (end not specified)	strong risk factors ^f^
**Canada (2018) [31]**	no recommendation		
**ACOG (2018) [84]**	81 mg	from 12 to delivery	≥1 high risk factor ^a^, or >1 moderate risk factor ^g^
**NICE (2019) [10]**	75–150 mg	from 12 to delivery	≥1 high risk factor ^d^, or >1 moderate risk factor ^e^
**Ireland (2019) [28]**	75–100 mg	from 12 to delivery	≥1 high risk factor ^d^, or >1 moderate risk factor ^e^
**FIGO (2019) [12]**	150 mg	from 11 to 14 to 36 weeks, or delivery, or preeclampsia	high risk (locally defined), or risk ≥1 in 100

Legend: ACOG (American College of Obstetricians and Gynecologists), ACC/AHA (American College of Cardiology/American Heart Association), Brazil (Brazilian Guideline of Arterial Hypertension), DGGG (Deutsche Gesellschaft für Gynäkologie und Geburtshilfe), ESC/ESH (European Society of Cardiology/European Society of Hypertension), FIGO (The International Federation of Gynecology and Obstetrics), Hypertension Canada, NICE (National Institute for Health and Care Excellence), Ireland (Royal College of Physicians of Ireland), SOMANZ (Society of Obstetric Medicine of Australia and New Zealand), Queensland (Queensland Clinical Guideline), USPSTF (US Preventive Services Task Force), WHO (World Health Organization). ^a^ High risk factors (ACOG, USPSTF, WHO): previous episode of preeclampsia, multiple gestations, chronic hypertension, type 1 or 2 diabetes mellitus, renal disease, autoimmune disease. ^b^ SOMANZ risk factors (no distinction between high or moderate risk): personal or family history of preeclampsia, nulliparity, multiple pregnancy, BMI ≥ 25 kg/m^2^, age ≥ 40, SBP > 130 mmHg or DBP > 80 mmHg before 20 gestational weeks, antiphospholipid syndrome, renal disease, pre-existing diabetes, chronic autoimmune disease, inter-pregnancy interval >10 years. ^c^ Queensland risk factors (no distinction between high or moderate risk): personal or family history of preeclampsia, multiple pregnancy (increased risk with multiples), nulliparity, pregestational BMI > 35 kg/m^2^, age > 40, SBP > 130 mmHg or DBP > 80 mmHg at initial visit, inter-pregnancy interval >10 years, renal disease, pre-existing diabetes, chronic autoimmune disease, chronic hypertension, antiphospholipid antibodies. ^d^ High risk factors (NICE, ESC/ESH, Royal College of Physicians of Ireland): a hypertensive disorder during a previous pregnancy, chronic kidney disease, autoimmune disease, pre-existing diabetes, chronic hypertension. ^e^ Moderate risk factors (NICE, ESC/ESH, Royal College of Physicians of Ireland): first pregnancy, age ≥ 40, pregnancy interval >10 years, BMI ≥ 35 kg/m^2^ at the first visit, family history of preeclampsia, multiple pregnancies. ^f^ ISSHP strong risk factors: history of preeclampsia, chronic hypertension, pre-existing diabetes, renal disease, maternal BMI > 30 kg/m^2^, multiple pregnancies, antiphospholipid syndrome, assisted reproduction. ^g^ Moderate risk factors (ACOG): nulliparity, obesity (BMI > 30 kg/m^2^), family history of preeclampsia (mother or sisters), African-American ethnicity, low socioeconomic status, age > 35, history of a neonate that is small for gestational age, previous adverse pregnancy outcome, or an inter-pregnancy interval of >10 years.

## Data Availability

Not applicable.
